# 2,4-Dibutoxy­phenyl­boronic acid

**DOI:** 10.1107/S1600536809023332

**Published:** 2009-06-24

**Authors:** Marek Dąbrowski, Sergiusz Luliński, Janusz Serwatowski, Agnieszka Wilmowicz

**Affiliations:** a Physical Chemistry Department, Faculty of Chemistry, Warsaw University of Technology, Noakowskiego 3, 00-664 Warsaw, Poland

## Abstract

In the crystal of the title compound, C_14_H_23_BO_4_, centrosymmetric dimers linked by pairs of O—H⋯O hydrogen bonds occur. The dimers are linked *via* C—H⋯O contacts, generating a two-dimensional array parallel to (12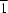). These are inter­connected by weak O—H⋯O hydrogen bonds, as well as C—H⋯π inter­actions.

## Related literature

For the structural characterization of related *ortho*-alk­oxy aryl­boronic acids, see: Dąbrowski *et al.* (2006 ▶, 2008 ▶); Luliński (2008 ▶); Yang *et al.* (2005 ▶).
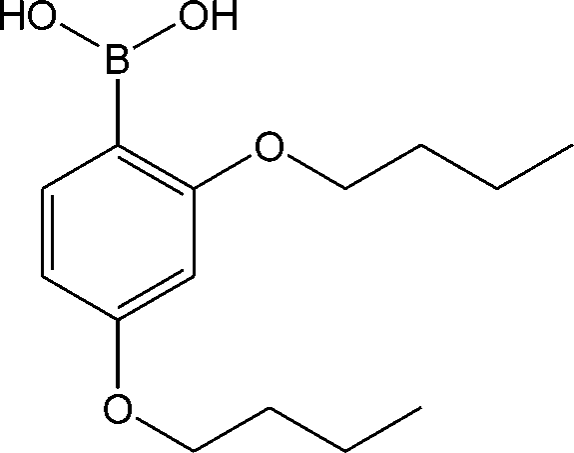


## Experimental

### 

#### Crystal data

C_14_H_23_BO_4_
*M* *_r_* = 266.13
Triclinic, 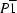

*a* = 5.3129 (8) Å
*b* = 11.3611 (13) Å
*c* = 13.7362 (17) Å
α = 112.747 (11)°
β = 94.311 (11)°
γ = 100.385 (11)°
*V* = 742.45 (17) Å^3^
*Z* = 2
Mo *K*α radiation
μ = 0.08 mm^−1^
*T* = 100 K
0.65 × 0.20 × 0.09 mm

#### Data collection

Oxford Diffraction KM-4-CCD diffractometer
Absorption correction: multi-scan (*CrysAlis RED*; Oxford Diffraction 2005 ▶) *T*_min_ = 0.96, *T*_max_ = 0.99
14053 measured reflections
3570 independent reflections
2633 reflections with *I* > 2σ(*I*)
*R*_int_ = 0.018

#### Refinement

*R*[*F* ^2^ > 2σ(*F* ^2^)] = 0.034
*wR*(*F* ^2^) = 0.095
*S* = 1.05
3570 reflections
180 parameters
H atoms treated by a mixture of independent and constrained refinement
Δρ_max_ = 0.38 e Å^−3^
Δρ_min_ = −0.23 e Å^−3^

### 

Data collection: *CrysAlis CCD* (Oxford Diffraction, 2005 ▶); cell refinement: *CrysAlis RED* (Oxford Diffraction, 2005 ▶); data reduction: *CrysAlis RED*; program(s) used to solve structure: *SHELXS97* (Sheldrick, 2008 ▶); program(s) used to refine structure: *SHELXL97* (Sheldrick, 2008 ▶); molecular graphics: *DIAMOND* (Brandenburg, 1999 ▶); software used to prepare material for publication: *SHELXL97*.

## Supplementary Material

Crystal structure: contains datablocks I, global. DOI: 10.1107/S1600536809023332/tk2482sup1.cif

Structure factors: contains datablocks I. DOI: 10.1107/S1600536809023332/tk2482Isup2.hkl

Additional supplementary materials:  crystallographic information; 3D view; checkCIF report

## Figures and Tables

**Table 1:** Hydrogen-bond geometry (Å, °)

*D*—H⋯*A*	*D*—H	H⋯*A*	*D*⋯*A*	*D*—H⋯*A*
O1—H1*o*⋯O2^i^	0.876 (16)	1.889 (17)	2.7649 (11)	178.9 (14)
O2—H2*o*⋯O3	0.839 (15)	2.129 (15)	2.7469 (10)	130.3 (13)
O2—H2*o*⋯O1^ii^	0.839 (15)	2.610 (15)	3.2205 (12)	130.7 (12)
C10—H10*C*⋯O4^iii^	0.98	2.60	3.5739 (15)	173 (1)
C9′—H9*B*′⋯O1^iv^	0.99	2.71	3.4314 (17)	130 (1)
C7—H7*B*⋯*Cg*^vi^	0.99	2.74	3.6237 (12)	149
C7′—H7*B*′⋯*Cg*^vii^	0.99	2.80	3.7109 (12)	153

Symmetry codes: (i) 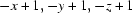; (ii) 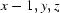; (iii) 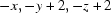; (iv) 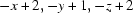; (v) 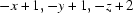; (vi) 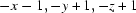; (vii) 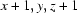. *Cg* is the centroid of the C1–C6 ring.

**Fig. 1.**
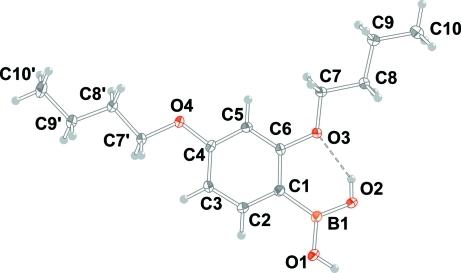
The molecular structure of (I) showing the atom-labelling scheme. The intramolecular hydrogen bond is shown as a dashed line. Displacement ellipsoids for all non-H atoms are drawn at the 50% probability level.

**Fig. 2.**
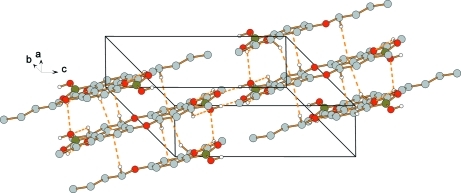
The crystal packing diagram for (I) showing hydrogen bonding and CH···π interactions (dashed lines). H atoms not involved in intermolecular interactions are omitted for clarity.

## supplementary crystallographic information

### Comment

The ability of arylboronic acids to form supramolecular assemblies due to intermolecular hydrogen bonding is well known. Our interest has focused on *ortho*-alkoxy derivatives and the influence of various factors (including the number and length of the alkoxy group) on their structural behaviour. The molecular structure of (I) shows the boronic groups possesses an *exo*-*endo* conformation and is slightly twisted with respect to the benzene ring, Fig. 1. However, the entire molecule including both butoxy groups remains essentially planar. The *endo*-oriented OH group is engaged in an intramolecular O—H···O hydrogen bond with the 2-butoxy-O atom, resulting in the formation of a six-membered ring, Table 1. This motif is generally typical of *ortho*-alkoxyarylboronic acids structures (Yang *et al.*, 2005; Dąbrowski *et al.*, 2006, 2008; Luliński, 2008). The supramolecular assembly of (I) is similar to that observed previously for 2,4-dimethoxyphenylboronic acid (Yang *et al.*, 2005). Centrosymmetric hydrogen-bonded dimers of (I) are linked by weaker C—H···O contacts, of which there are two types (Table 1). The first one connects the terminal methyl of the 2-butoxy group with the O atom of the adjacent molecule whereas the second one is formed between the γ-methylene unit of the 4-butoxy group and the O atom of the *exo*-OH group. As a result a 2-D array is formed, aligned parallel to the (121) plane. The supramolecular architecture extends further due to other interactions. They involve weak cross-linking O—H···O hydrogen bonds between the *endo*-OH group and the O atom of *exo*-OH group from the neighbouring layer. Finally, C—H···π interactions occur between the α-methylene units of 2-butoxy as well as 4-butoxy groups and the aromatic ring of another molecule; the distances of H7B and H7B' from the ring centroid are 2.7363 (4)Å [symmetry code (vi): -1 - *x*, 1 - *y*, 1 - *z*] and 2.8035 (5)Å [symmetry code (vii): 1 + *x*, *y*, 1 + *z*], respectively. As a result, a 3-D network is formed. In conclusion, the hydrogen-bonded dimeric structure of (I) is typical of boronic acids whereas the unique secondary supramolecular assembly is achieved due to weaker hydrogen bonds and C—H-π interactions.

### Experimental

Crystals were grown by slow evaporation of a hexane/acetone (10 ml, 1:1) solution of (I) (Aldrich, 0.3 g).

### Refinement

Carbon-bound H atoms were placed in calculated positions with C—H = 0.95–0.99 Å, and were included in the refinement in the riding model approximation with *U*(H) set to 1.2–1.5*U*_eq_(C). The H1o and H2o atoms were refined without constraint; O—H distances = 0.839 (15) and 0.876 (16) Å.

### Crystal data

**Table:**

C_14_H_23_BO_4_	*V* = 742.45 (17) Å^3^
*M**_r_* = 266.13	*Z* = 2
Triclinic, *P*1	*F*(000) = 288
Hall symbol: -P 1	*D*_x_ = 1.190 Mg m^−^^3^
*a* = 5.3129 (8) Å	Melting point: 369 K
*b* = 11.3611 (13) Å	Mo *K*α radiation, λ = 0.71073 Å
*c* = 13.7362 (17) Å	µ = 0.08 mm^−^^1^
α = 112.747 (11)°	*T* = 100 K
β = 94.311 (11)°	Prismatic, colourless
γ = 100.385 (11)°	0.65 × 0.20 × 0.09 mm

### Data collection

**Table:**

Oxford Diffraction KM-4-CCD diffractometer	3570 independent reflections
Radiation source: fine-focus sealed tube	2633 reflections with *I* > 2σ(*I*)
graphite	*R*_int_ = 0.018
Detector resolution: 8.6479 pixels mm^-1^	θ_max_ = 28.6°, θ_min_ = 3.1°
ω scans	*h* = −7→7
Absorption correction: multi-scan (*CrysAlis RED*; Oxford Diffraction 2005)	*k* = −15→15
*T*_min_ = 0.96, *T*_max_ = 0.99	*l* = −18→18
14053 measured reflections	

### Refinement

**Table:**

Refinement on *F*^2^	Primary atom site location: structure-invariant direct methods
Least-squares matrix: full	Secondary atom site location: difference Fourier map
*R*[*F*^2^ > 2σ(*F*^2^)] = 0.034	Hydrogen site location: inferred from neighbouring sites
*wR*(*F*^2^) = 0.095	H atoms treated by a mixture of independent and constrained refinement
*S* = 1.05	*w* = 1/[σ^2^(*F*_o_^2^) + (0.0557*P*)^2^] where *P* = (*F*_o_^2^ + 2*F*_c_^2^)/3
3570 reflections	(Δ/σ)_max_ = 0.001
180 parameters	Δρ_max_ = 0.38 e Å^−^^3^
0 restraints	Δρ_min_ = −0.23 e Å^−^^3^

### Special details

**Table:**

Geometry. All e.s.d.'s (except the e.s.d. in the dihedral angle between two l.s. planes) are estimated using the full covariance matrix. The cell e.s.d.'s are taken into account individually in the estimation of e.s.d.'s in distances, angles and torsion angles; correlations between e.s.d.'s in cell parameters are only used when they are defined by crystal symmetry. An approximate (isotropic) treatment of cell e.s.d.'s is used for estimating e.s.d.'s involving l.s. planes.
Refinement. Refinement of *F*^2^ against ALL reflections. The weighted *R*-factor *wR* and goodness of fit *S* are based on *F*^2^, conventional *R*-factors *R* are based on *F*, with *F* set to zero for negative *F*^2^. The threshold expression of *F*^2^ > σ(*F*^2^) is used only for calculating *R*-factors(gt) *etc*. and is not relevant to the choice of reflections for refinement. *R*-factors based on *F*^2^ are statistically about twice as large as those based on *F*, and *R*- factors based on ALL data will be even larger.

### Fractional atomic coordinates and isotropic or equivalent isotropic displacement parameters (Å^2^)

**Table:**

	*x*	*y*	*z*	*U*_iso_*/*U*_eq_	
C1	0.56253 (19)	0.61272 (9)	0.78929 (7)	0.0153 (2)	
C2	0.72694 (19)	0.56726 (10)	0.84327 (8)	0.0170 (2)	
H2	0.8262	0.5095	0.8031	0.020*	
C3	0.75323 (19)	0.60219 (10)	0.95310 (8)	0.0171 (2)	
H3	0.8666	0.5685	0.9869	0.021*	
C4	0.60985 (19)	0.68740 (10)	1.01210 (7)	0.0155 (2)	
C5	0.44559 (18)	0.73795 (9)	0.96259 (8)	0.0159 (2)	
H5	0.3502	0.7974	1.0036	0.019*	
C6	0.42293 (18)	0.70058 (9)	0.85288 (8)	0.0146 (2)	
C7	0.14424 (19)	0.84955 (10)	0.85803 (8)	0.0167 (2)	
H7A	0.2754	0.9274	0.9086	0.020*	
H7B	0.0232	0.8190	0.8992	0.020*	
C8	−0.00127 (19)	0.88312 (10)	0.77716 (8)	0.0169 (2)	
H8A	−0.1220	0.8025	0.7244	0.020*	
H8B	0.1239	0.9154	0.7382	0.020*	
C9	−0.15422 (19)	0.98636 (10)	0.82756 (8)	0.0187 (2)	
H9A	−0.0328	1.0699	0.8751	0.022*	
H9B	−0.2692	0.9580	0.8717	0.022*	
C10	−0.3170 (2)	1.00821 (11)	0.74256 (9)	0.0240 (2)	
H10A	−0.4393	0.9259	0.6962	0.036*	
H10B	−0.2032	1.0378	0.6996	0.036*	
H10C	−0.4132	1.0750	0.7772	0.036*	
C7'	0.79357 (19)	0.68855 (10)	1.17808 (8)	0.0173 (2)	
H7A'	0.9734	0.7183	1.1687	0.021*	
H7B'	0.7528	0.5918	1.1514	0.021*	
C8'	0.7655 (2)	0.74945 (10)	1.29481 (8)	0.0192 (2)	
H8A'	0.5885	0.7140	1.3037	0.023*	
H8B'	0.7892	0.8454	1.3185	0.023*	
C9'	0.9634 (2)	0.72152 (11)	1.36433 (8)	0.0244 (3)	
H9A'	1.1400	0.7584	1.3560	0.029*	
H9B'	0.9421	0.6255	1.3391	0.029*	
C10'	0.9371 (3)	0.77900 (13)	1.48245 (9)	0.0378 (3)	
H10D	0.7640	0.7414	1.4915	0.057*	
H10E	0.9621	0.8743	1.5085	0.057*	
H10F	1.0684	0.7582	1.5233	0.057*	
O1	0.73656 (14)	0.51449 (8)	0.61804 (6)	0.0245 (2)	
O2	0.34414 (15)	0.57844 (8)	0.60251 (6)	0.0253 (2)	
O3	0.26778 (13)	0.74719 (7)	0.79858 (5)	0.01810 (17)	
O4	0.61551 (13)	0.72922 (7)	1.12026 (5)	0.01908 (18)	
B1	0.5445 (2)	0.56749 (11)	0.66526 (9)	0.0170 (2)	
H1o	0.709 (3)	0.4847 (14)	0.5482 (13)	0.049 (4)*	
H2o	0.231 (3)	0.6103 (14)	0.6371 (12)	0.051 (4)*	

### Atomic displacement parameters (Å^2^)

**Table:**

	*U*^11^	*U*^22^	*U*^33^	*U*^12^	*U*^13^	*U*^23^
C1	0.0172 (5)	0.0154 (5)	0.0130 (5)	0.0038 (4)	0.0023 (4)	0.0053 (4)
C2	0.0199 (5)	0.0166 (5)	0.0144 (5)	0.0070 (4)	0.0038 (4)	0.0049 (4)
C3	0.0201 (5)	0.0188 (5)	0.0150 (5)	0.0080 (4)	0.0012 (4)	0.0083 (4)
C4	0.0180 (5)	0.0172 (5)	0.0106 (5)	0.0029 (4)	0.0018 (4)	0.0057 (4)
C5	0.0166 (5)	0.0166 (5)	0.0140 (5)	0.0060 (4)	0.0034 (4)	0.0045 (4)
C6	0.0140 (5)	0.0160 (5)	0.0149 (5)	0.0036 (4)	0.0005 (4)	0.0077 (4)
C7	0.0188 (5)	0.0176 (5)	0.0137 (5)	0.0084 (4)	0.0033 (4)	0.0045 (4)
C8	0.0179 (5)	0.0190 (5)	0.0155 (5)	0.0068 (4)	0.0027 (4)	0.0078 (4)
C9	0.0197 (5)	0.0185 (5)	0.0188 (5)	0.0073 (4)	0.0044 (4)	0.0071 (4)
C10	0.0232 (6)	0.0251 (6)	0.0264 (6)	0.0108 (5)	0.0040 (4)	0.0111 (5)
C7'	0.0198 (5)	0.0211 (5)	0.0133 (5)	0.0087 (4)	0.0019 (4)	0.0079 (4)
C8'	0.0244 (6)	0.0222 (5)	0.0126 (5)	0.0093 (4)	0.0038 (4)	0.0069 (4)
C9'	0.0322 (6)	0.0298 (6)	0.0145 (5)	0.0136 (5)	0.0032 (4)	0.0097 (5)
C10'	0.0571 (9)	0.0448 (8)	0.0151 (6)	0.0230 (7)	0.0018 (5)	0.0117 (5)
O1	0.0283 (4)	0.0359 (5)	0.0108 (4)	0.0177 (4)	0.0041 (3)	0.0064 (3)
O2	0.0296 (4)	0.0386 (5)	0.0122 (4)	0.0227 (4)	0.0060 (3)	0.0081 (3)
O3	0.0230 (4)	0.0218 (4)	0.0117 (3)	0.0131 (3)	0.0022 (3)	0.0057 (3)
O4	0.0248 (4)	0.0248 (4)	0.0107 (3)	0.0120 (3)	0.0029 (3)	0.0076 (3)
B1	0.0229 (6)	0.0161 (6)	0.0125 (5)	0.0067 (5)	0.0026 (5)	0.0053 (4)

### Geometric parameters (Å, °)

**Table:**

C1—C2	1.3932 (13)	C9—H9B	0.9900
C1—C6	1.4140 (13)	C10—H10A	0.9800
C1—B1	1.5674 (14)	C10—H10B	0.9800
C2—C3	1.3923 (13)	C10—H10C	0.9800
C2—H2	0.9500	C7'—O4	1.4379 (11)
C3—C4	1.3879 (14)	C7'—C8'	1.5123 (14)
C3—H3	0.9500	C7'—H7A'	0.9900
C4—O4	1.3697 (11)	C7'—H7B'	0.9900
C4—C5	1.3955 (13)	C8'—C9'	1.5237 (14)
C5—C6	1.3875 (13)	C8'—H8A'	0.9900
C5—H5	0.9500	C8'—H8B'	0.9900
C6—O3	1.3736 (11)	C9'—C10'	1.5251 (15)
C7—O3	1.4392 (11)	C9'—H9A'	0.9900
C7—C8	1.5105 (13)	C9'—H9B'	0.9900
C7—H7A	0.9900	C10'—H10D	0.9800
C7—H7B	0.9900	C10'—H10E	0.9800
C8—C9	1.5217 (13)	C10'—H10F	0.9800
C8—H8A	0.9900	O1—B1	1.3577 (13)
C8—H8B	0.9900	O1—H1o	0.876 (16)
C9—C10	1.5241 (14)	O2—B1	1.3727 (13)
C9—H9A	0.9900	O2—H2o	0.839 (15)
			
C2—C1—C6	116.20 (8)	C9—C10—H10B	109.5
C2—C1—B1	119.80 (8)	H10A—C10—H10B	109.5
C6—C1—B1	123.99 (9)	C9—C10—H10C	109.5
C3—C2—C1	123.36 (9)	H10A—C10—H10C	109.5
C3—C2—H2	118.3	H10B—C10—H10C	109.5
C1—C2—H2	118.3	O4—C7'—C8'	107.43 (8)
C4—C3—C2	118.41 (9)	O4—C7'—H7A'	110.2
C4—C3—H3	120.8	C8'—C7'—H7A'	110.2
C2—C3—H3	120.8	O4—C7'—H7B'	110.2
O4—C4—C3	124.59 (9)	C8'—C7'—H7B'	110.2
O4—C4—C5	114.68 (8)	H7A'—C7'—H7B'	108.5
C3—C4—C5	120.73 (9)	C7'—C8'—C9'	111.40 (8)
C6—C5—C4	119.37 (9)	C7'—C8'—H8A'	109.3
C6—C5—H5	120.3	C9'—C8'—H8A'	109.3
C4—C5—H5	120.3	C7'—C8'—H8B'	109.3
O3—C6—C5	122.81 (8)	C9'—C8'—H8B'	109.3
O3—C6—C1	115.27 (8)	H8A'—C8'—H8B'	108.0
C5—C6—C1	121.91 (8)	C8'—C9'—C10'	112.88 (9)
O3—C7—C8	106.65 (8)	C8'—C9'—H9A'	109.0
O3—C7—H7A	110.4	C10'—C9'—H9A'	109.0
C8—C7—H7A	110.4	C8'—C9'—H9B'	109.0
O3—C7—H7B	110.4	C10'—C9'—H9B'	109.0
C8—C7—H7B	110.4	H9A'—C9'—H9B'	107.8
H7A—C7—H7B	108.6	C9'—C10'—H10D	109.5
C7—C8—C9	113.05 (8)	C9'—C10'—H10E	109.5
C7—C8—H8A	109.0	H10D—C10'—H10E	109.5
C9—C8—H8A	109.0	C9'—C10'—H10F	109.5
C7—C8—H8B	109.0	H10D—C10'—H10F	109.5
C9—C8—H8B	109.0	H10E—C10'—H10F	109.5
H8A—C8—H8B	107.8	B1—O1—H1o	113.8 (9)
C8—C9—C10	111.28 (8)	B1—O2—H2o	113.1 (10)
C8—C9—H9A	109.4	C6—O3—C7	119.23 (7)
C10—C9—H9A	109.4	C4—O4—C7'	117.93 (7)
C8—C9—H9B	109.4	O1—B1—O2	118.73 (9)
C10—C9—H9B	109.4	O1—B1—C1	118.07 (9)
H9A—C9—H9B	108.0	O2—B1—C1	123.20 (9)
C9—C10—H10A	109.5		
			
C6—C1—C2—C3	1.25 (15)	C7—C8—C9—C10	−174.85 (9)
B1—C1—C2—C3	−179.69 (9)	O4—C7'—C8'—C9'	−175.14 (8)
C1—C2—C3—C4	−0.48 (15)	C7'—C8'—C9'—C10'	−178.76 (10)
C2—C3—C4—O4	179.58 (9)	C5—C6—O3—C7	6.91 (13)
C2—C3—C4—C5	−0.72 (15)	C1—C6—O3—C7	−172.13 (8)
O4—C4—C5—C6	−179.21 (8)	C8—C7—O3—C6	177.59 (8)
C3—C4—C5—C6	1.07 (15)	C3—C4—O4—C7'	3.91 (14)
C4—C5—C6—O3	−179.22 (9)	C5—C4—O4—C7'	−175.81 (8)
C4—C5—C6—C1	−0.24 (14)	C8'—C7'—O4—C4	178.89 (8)
C2—C1—C6—O3	178.18 (8)	C2—C1—B1—O1	−17.36 (14)
B1—C1—C6—O3	−0.84 (14)	C6—C1—B1—O1	161.62 (10)
C2—C1—C6—C5	−0.88 (14)	C2—C1—B1—O2	162.36 (10)
B1—C1—C6—C5	−179.89 (9)	C6—C1—B1—O2	−18.66 (16)
O3—C7—C8—C9	177.25 (8)		

### Hydrogen-bond geometry (Å, °)

**Table:**

*D*—H···*A*	*D*—H	H···*A*	*D*···*A*	*D*—H···*A*
O1—H1o···O2^i^	0.876 (16)	1.889 (17)	2.7649 (11)	178.9 (14)
O2—H2o···O3	0.839 (15)	2.129 (15)	2.7469 (10)	130.3 (13)
O2—H2o···O1^ii^	0.839 (15)	2.610 (15)	3.2205 (12)	130.7 (12)
C10—H10C···O4^iii^	0.98	2.60	3.5739 (15)	173 (1)
C9'—H9B'···O1^iv^	0.99	2.71	3.4314 (17)	130 (1)
C7—H7B···Cg^v^	0.99	2.74	3.6237 (12)	149
C7'—H7B'···Cg^vi^	0.99	2.80	3.7109 (12)	153

Symmetry codes: (i) −*x*+1, −*y*+1, −*z*+1; (ii) *x*−1, *y*, *z*; (iii) −*x*, −*y*+2, −*z*+2; (iv) −*x*+2, −*y*+1, −*z*+2; (v) −*x*−1, −*y*+1, −*z*+1; (vi) *x*+1, *y*, *z*+1.
